# Anesthesia Considerations for Transfemoral Osseointegration in a Patient With CRPS, Propofol, and Ketamine Allergies: A Case Report

**DOI:** 10.1155/cria/6532945

**Published:** 2026-10-21

**Authors:** Jake D. Fanizza, Jason Shih Hoellwarth, Uchenna O. Umeh

**Affiliations:** ^1^ College of Medicine Drexel University Philadelphia Pennsylvania USA drexel.edu; ^2^ Osseointegration Limb Replacement Center Hospital for Special Surgery New York New York USA hss.edu; ^3^ Department of Anesthesiology Weill Cornell Medical College New York New York USA cornell.edu; ^4^ Department of Anesthesiology, Critical Care and Pain Management Hospital for Special Surgery New York New York USA hss.edu

**Keywords:** case report, chronic regional pain syndrome, neuraxial anesthesia, osseointegration, regional anesthesia

## Abstract

A 20‐year‐old female with asthma, Ehlers–Danlos syndrome, Hashimoto’s thyroiditis, mast cell activation syndrome, and complex regional pain syndrome Type 1 (CRPS1) presented for left transfemoral amputation with osseointegration and nerve reconstruction. To limit CRPS1 exacerbation, neuraxial anesthesia with femoral and sciatic blocks was administered for analgesia. Given the reported history of convulsions following ketamine and propofol, sedation with midazolam, dexmedetomidine, and fentanyl was used. On POD 1, fascia iliaca and sciatic blocks were repeated to mitigate postoperative pain. There were no intraoperative or postoperative complications. With limited literature on anesthesia for osseointegration, this novel case highlights individualized planning, in the setting of adverse responses to anesthetic medications and challenging pain management.

## 1. Introduction

Complex regional pain syndrome Type 1 (CRPS1) is a debilitating condition characterized by disproportionate sensory, motor, vasomotor, and trophic changes in a regional distribution [1]. In cases refractory to conservative treatments, amputation offers high patient satisfaction rates with improvements in quality of life. However, amputation for CRPS1 has low rates of prosthesis compatibility with postoperative complications including phantom limb pain, residual limb pain, and CRPS1 recurrence [2].

Osseointegration surgery [3, 4] offers excellent functional outcomes, improved mobility, and adequate pain control for patients with CRPS1 undergoing lower extremity amputation [5]. This innovative procedure places an implant such as a titanium transcutaneous osseointegration nail (TiTON) attachment into the residual bone to create a direct connection between the skeletal system and external prosthesis. Nevertheless, this intervention presents unique anesthetic challenges particularly in high‐risk patients.

Analgesia planning for lower extremity amputation often includes neuraxial anesthesia with peripheral nerve blocks (PNBs) as this is associated with reduced pain scores, hospital length of stay, and complications [6]. Furthermore, ketamine and propofol have shown to be effective for sedation in osseointegration [7]. However, with limited literature describing anesthesia planning or alternative regimens for patients with multiple comorbidities and contraindications to traditional agents, careful perioperative planning is crucial.

Here, we describe our anesthesia planning for osseointegration in a young patient with complex neurologic considerations and suspected adverse reactions to routine medications. Utilizing dexmedetomidine, midazolam, and fentanyl sedation, combined with neuraxial and PNBs, we provided safe and effective anesthesia.

Written Health Insurance Portability and Accountability Act (HIPPA) Authorization was obtained, and this report adheres to EQUATOR CARE guidelines.

## 2. Case Description

A 20‐year‐old female with CRPS1, asthma, mast cell activation syndrome, Hashimoto’s thyroiditis, Ehlers–Danlos syndrome, and convulsions following administration of ketamine and propofol presented for elective left transfemoral amputation with osseointegration and sciatic regenerative peripheral nerve interface (RPNI). Her orthopedic history was all provided elsewhere and began five years prior (2020) with treatment of hip dysplasia via hip arthroscopy and periacetabular osteotomy, complicated by femoral and sciatic nerve palsies and subsequent development of CRPS1 of the left lower extremity (Figure 1). Persistent neuropathic pain and functional decline resulted in wheelchair dependence. A spinal cord stimulator was placed and later removed due to inadequate relief. She underwent left total hip replacement in 2023, complicated by urinary retention requiring suprapubic catheter placement. Following several of the surgeries, she exhibited convulsions after exposure to propofol and ketamine. Neurologic workup including electroencephalography did not identify seizure activity.

**FIGURE 1:**
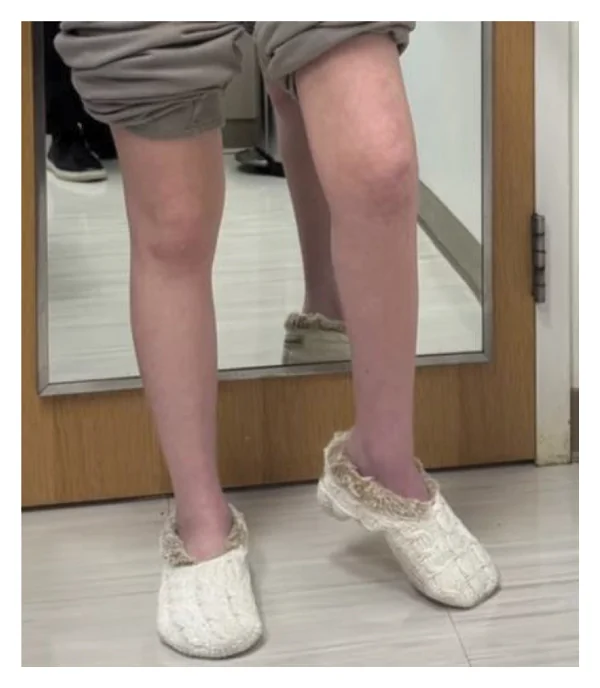
Clinical photograph prior to surgery, identifying the patient to have left lower extremity violaceous color, inability to stand on the leg, swelling, and disuse atrophy.

Given her complex neurologic history, chronic pain condition, and anesthetic drug reactions, a plan for neuraxial anesthetic with PNBs was developed to minimize central sensitization, avoid seizure‐provoking agents, seizures, and manage postoperative pain. The case required careful multidisciplinary planning to balance surgical requirements with significant anesthetic limitations. The primary goals were avoidance of seizure‐triggering agents, mitigation of CRPS exacerbation, maintenance of hemodynamic stability, and optimization of postoperative analgesia.

With the reported history of convulsions following ketamine and propofol exposure, general anesthesia was avoided. Additionally, minimizing central sensitization was considered critical in the setting of CRPS1. Neuraxial anesthesia was, therefore, selected as the primary anesthetic modality. A spinal anesthetic with bupivacaine 0.5% 3 mL was administered to provide surgical anesthesia while avoiding airway manipulation and reducing systemic anesthetic exposure. Ultrasound‐guided femoral (Figure 2) and sciatic nerve blocks with bupivacaine 0.25% were placed after the spinal.

**FIGURE 2:**
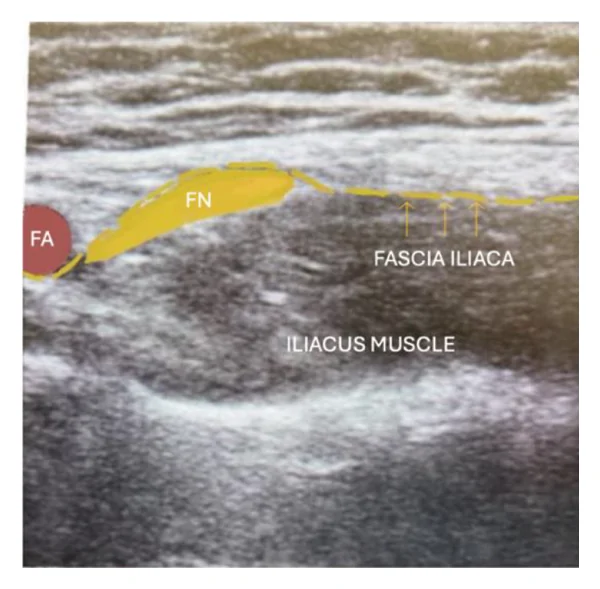
Image of ultrasound‐guided femoral nerve block. FA, femoral artery; FN, femoral nerve.

Intraoperative sedation was maintained using a dexmedetomidine infusion supplemented with intermittent boluses of midazolam and fentanyl. Dexmedetomidine was selected for its sedative and analgesic properties without lowering seizure threshold and for its minimal respiratory depression. Benzodiazepine supplementation provided anxiolysis and seizure prophylaxis, while opioid boluses were used judiciously for intraoperative stimulation.

Given the patient’s chronic neuropathic pain condition, a multimodal analgesia strategy was implemented for postoperative analgesia. Ultrasound‐guided femoral and infragluteal sciatic nerve blocks were performed in the operating room prior to surgical incision. The blocks were selected to reduce acute postoperative pain, decrease opioid requirements, and potentially reduce risk of CRPS exacerbation. Hemodynamics remained stable throughout the procedure, and no seizure activity or anesthetic complications occurred. The avoidance of general anesthesia and seizure‐triggering agents, combined with targeted peripheral nerve blockade, allowed for effective surgical anesthesia and optimized postoperative pain control in a complex patient.

The patient underwent neuraxial anesthesia and the surgical procedure without intraoperative complications. Sensory and motor blockade was adequate for the duration of the case, and sedation with dexmedetomidine infusion provided adequate sedation without respiratory compromise. No convulsive or seizure activity or hemodynamic instability was observed. She was transferred to the postanesthesia care unit hemodynamically stable, neurologically appropriate, and comfortable.

Postoperatively, pain scores in the immediate period were well controlled; we attribute this to the combined neuraxial and peripheral nerve blockade strategy. Importantly, no acute CRPS flare symptoms occurred. There was no evidence of urinary retention, respiratory depression, or neurologic deterioration. The perioperative pain service (POPS) was consulted given her pain history and medication reactions.

On postoperative day one, with block resolution, she complained of increasing severe pain. The decision was made to place single shot fascia iliaca and sciatic PNBs, and the patient’s pain remained manageable with a multimodal analgesia protocol which included hydromorphone, acetaminophen, NSAIDS, pregabalin, methocarbamol, and lidocaine patches. No convulsions or seizure activity occurred during hospitalization. The patient began postoperative rehabilitation planning focused on future prosthetic integration.

At her 6‐week follow‐up, the patient demonstrated stable wound healing without infection or implant malposition. Pain remained controlled without evidence of CRPS1 exacerbation. There were no subsequent seizures or anesthesia‐related complications. Radiographic evaluation confirmed appropriate positioning of the TiTON implant without hardware failure or periprosthetic complication (Figure 3). The patient was fit for her prosthetic leg at 3 months and has remained independently ambulatory, 8 months postoperation.

**FIGURE 3:**
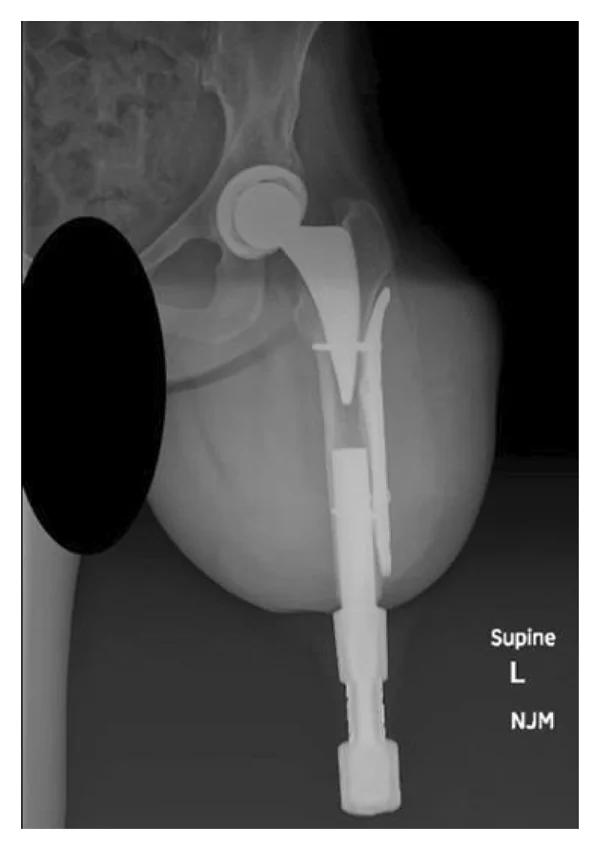
Six‐week postoperative left femur anterior–posterior radiograph showing the preexisting total hip replacement, with new TiTON implant, and prophylactic plate bridging the two implants to protect against potential future interprosthetic fracture.

## 3. Discussion

Osseointegration for refractory CRPS1 offers clinically significant improvements in function and quality of life including pain‐free ambulation in patients who were previously wheelchair dependent [5]. Despite these benefits, literature outlining anesthesia and pain management for osseointegration is limited particularly for patients with multiple allergy considerations. In this case, we describe important anesthesia considerations and the navigation of propofol and ketamine adverse reactions to deliver optimal anesthesia care for osseointegration in CRPS.

A meta‐analysis conducted by Mufarrih and colleagues showed that in lower extremity amputation, patients who received regional anesthesia experienced lower rates of sepsis and respiratory complications compared to general anesthesia [8]. Similarly, Huh and colleagues’ retrospective study showed regional anesthesia to have significantly lower rates of postoperative pain, length of stay, and delirium [6]. While current literature does not describe regional anesthesia in osseointegration, this evidence guided our decision to utilize neuraxial anesthesia and repeat fascia iliaca and sciatic blocks to limit postoperative pain and optimize outcomes.

Furthermore, patients with CRPS require diligent pain management planning as they are prone to hyperalgesia and central sensitization [9]. Surgical procedures may be an aggravating factor of CRPS exacerbation, but some data have demonstrated that PNB can mitigate this risk [10]. One retrospective study found that in patients with CRPS undergoing surgery, 10% of those who received regional blockade, including ganglion block, versus 72% who did not, experienced CRPS exacerbation [11]. This finding supports our decision to perform PNBs as they may inhibit afferent triggering signals and subsequent sympathetic pain response. Additionally, our repeat administration of spinal anesthesia and postoperative PNBs illustrates the importance of coordinated intervention, and the potentially increased analgesic needs in this population. The multimodal pain approach achieved avoiding acute CRPS flare, postoperative complications, and chronic pain for this individual patient.

Avoidance of ketamine, propofol, and epileptic agents were the main challenge in providing this patient’s anesthesia. Chattopadhyay et al. demonstrated that dexmedetomidine can safely function as a sole maintenance agent while others have highlighted its anti‐inflammatory and respiratory preserving properties [12, 13]. Further, randomized trials have demonstrated that compared to propofol, dexmedetomidine provides similar sedation and may offer superior hemodynamic control and reduced postoperative opioids requirements [12–14]. This evidence guided our choice of dexmedetomidine alongside adjuvant fentanyl and midazolam. Our use of dexmedetomidine provided a valuable alternative to traditional methods for this patient’s care.

The anesthetic care for patients undergoing osseointegration is extremely limited, with only one article providing substantive information [7]. Additionally, there is only one paper describing osseointegration for CRPS patients [5]. The anesthetic considerations for patients undergoing osseointegration are likely very similar to patients undergoing amputation surgery; the anatomic location is identical. However, considerations such as the potential circulatory and pain‐related impacts of reaming the intramedullary canal and placing an implant into the bone have not been specifically compared, so may be different. In particular, CRPS is very poorly understood, including for surgical amputation, let alone with osseointegration. The anesthetic considerations for intraoperative, immediate postoperative, and long‐term postoperative pain relief or exacerbation in patients undergoing osseointegration have been very minimally explored.

This case report has several limitations. While our analgesic technique provided adequate postoperative pain control and may have aided in prevention of CRPS exacerbation, it is not possible to determine which individual interventions may have contributed to this favorable outcome. Clinicians should be cautious in generalizing from this case as our protocol was selected based on this patient’s unique medical history and medication responses.

## 4. Conclusion

This case demonstrates that, for this specific patient, neuraxial anesthesia combined with peripheral nerve blockade achieved stable intraoperative conditions and effective postoperative analgesia while avoiding anesthetic agents that were associated with adverse responses for her. This demonstrates that this strategy can be an effective primary technique for osseointegration in patients with concerns regarding common induction agents.

## Funding

No funding was received for this manuscript.

## Conflicts of Interest

The authors declare no conflicts of interest.

## Supporting Information

Additional supporting information can be found online in the Supporting Information section.

## Supporting information


**Supporting Information** CARE checklist.

## Data Availability

The data that support the findings of this study are available from the corresponding author upon reasonable request.
